# Pustular reaction in adult-onset immunodeficiency due to anti-interferon-gamma autoantibodies

**DOI:** 10.3389/fimmu.2025.1619832

**Published:** 2025-08-22

**Authors:** Narachai Julanon, Pojsakorn Danpanichkul, Charoen Choonhakarn, Suteeraporn Chaowattanapanit, Siriluck Anunnatsiri, Ploenchan Chetchotisakd, Salin Kiratikanon, Rujira Rujiwetpongstorn, Napatra Tovanabutra, Siri Chiewchanvit, Romanee Chaiwarith, Phichayut Phinyo, Mati Chuamanochan

**Affiliations:** ^1^ Division of Dermatology, Department of Medicine, Khon Kaen University, Khon Kaen, Thailand; ^2^ Department of Internal Medicine, Texas Tech University Health Sciences Center, Lubbock, TX, United States; ^3^ Division of Infectious Disease and Tropical Medicine, Department of Medicine, Khon Kaen University, Khon Kaen, Thailand; ^4^ Division of Dermatology, Department of Internal Medicine, Faculty of Medicine, Chiang Mai University, Chiang Mai, Thailand; ^5^ Division of Infectious Diseases and Tropical Medicine, Department of Internal Medicine, Faculty of Medicine, Chiang Mai University, Chiang Mai, Thailand; ^6^ Center for Clinical Epidemiology and Clinical Statistics, Faculty of Medicine, Chiang Mai University, Chiang Mai, Thailand; ^7^ Department of Biomedical Informatics and Clinical Epidemiology, Faculty of Medicine, Chiang Mai University, Chiang Mai, Thailand; ^8^ Pharmacoepidemiology and Statistics Research Center (PESRC), Faculty of Pharmacy, Chiang, Mai University, Chiang Mai, Thailand

**Keywords:** acute generalized exanthematous pustulosis, adult-onset immunodeficiency, anti-interferongamma autoantibodies, pustular psoriasis, pustular reaction

## Abstract

**Introduction:**

Cutaneous manifestations in adult-onset immunodeficiency (AOID) resulting from anti-interferon-gamma autoantibody (AIGA) are prevalent and can be classified into infective and reactive disorders. To date, no clinical studies have specifically examined pustular reaction in patients with AOID. This study aimed to provide an original characterization of the clinical manifestations associated with pustular reaction in AOID and to compare these features with those observed in a clinically similar entity, generalized pustular psoriasis (GPP).

**Methods:**

A retrospective study was conducted between January 2014 and June 2023 at the dermatology clinics of Maharaj Nakorn Chiang Mai Hospital, Chiang Mai and Srinagarind Hospital, Khon Kaen, Thailand. The study included adult patients diagnosed with AOID presenting with pustular reaction, defined as pinhead-sized pustules on an erythematous base occurring any area of skin, as well as those diagnosed with GPP. Cases with pustular drug eruption and with non-sterile pustules were excluded. Data analysis was performed using both univariable and multivariable statistical methods.

**Results:**

Total of 64 patients diagnosed with AOID who subsequently developed a pustular reaction were included in the study. Clinically, the cutaneous manifestations were characterized by discrete, pinhead-sized pustules distributed on erythematous bases, predominantly affecting the trunk and extremities. A concomitant infection was identified in 54 patients (84.4%). Rapid-growing mycobacteria represented the most frequently identified pathogens, with lymph nodes being the most commonly involved anatomical site. Comparative analysis with a cohort of 77 patients diagnosed with GPP elucidated distinct clinical hallmarks differentiating the two entities. Lymphadenopathy, hepatomegaly, and splenomegaly emerged as distinguishing features more frequently associated with AOID-related pustular reaction. In contrast, the presence of geographic tongue and nail involvement was more characteristic of GPP. In instances where these pathognomonic features were absent, a multivariable predictive model was developed to aid in diagnostic differentiation. This model incorporated the presence of concomitant infections, elevated serum globulin levels, and increased alkaline phosphatase levels.

**Conclusion:**

Among patients presenting with sterile pustular eruptions, the presence of lymphadenopathy, hepatomegaly, and splenomegaly served as perfect clinical predictors of an underlying pustular reaction associated with AOID. In cases where these hallmark features were absent, a predictive algorithm incorporating the presence of a concomitant infections, serum globulin concentration, and alkaline phosphatase level demonstrated robust utility in estimating the likelihood of AOID-associated pustular reaction.

## 1 Introduction

Adult-onset immunodeficiency (AOID) due to anti-interferon-gamma autoantibodies (AIGA) is an increasingly recognized form of acquired immunodeficiency, frequently associated with cutaneous manifestations (1, 2). AOID is characterized by the presence of high-titer, neutralizing autoantibodies directed against interferon-gamma (IFN-γ), a key cytokine in host defense against intracellular pathogens. The condition primarily affects individuals of Southeast and East Asian descent, implicating potential genetic predisposition in its pathogenesis (1, 3). Nevertheless, the typical age of onset—around the fifth decade of life—suggests that environmental factors may also contribute significantly to disease development.

The functional impairment of IFN-γ signaling results in marked susceptibility to opportunistic infections (OIs), mirroring the immunological deficits observed in acquired immunodeficiency syndrome (AIDS). The most frequently encountered OIs in AOID include non-tuberculous mycobacteria (NTM), dimorphic fungi, and non-typhoidal *Salmonella* species. Lymph nodes are the most commonly affected anatomical sites, followed by pulmonary, osseous, and cutaneous involvement (4).

Cutaneous manifestations are not uncommon in AOID and may serve as important clinical clues to the underlying immunologic defect. These dermatologic findings can be broadly classified into two categories: infectious and reactive dermatoses. Infectious skin conditions include viral infections such as herpes simplex and varicella-zoster virus, cutaneous involvement by NTM, and invasive dimorphic fungal infections, notably *Talaromyces marneffei* and *Histoplasma capsulatum* (5). Among the reactive dermatoses, neutrophilic dermatoses are the most prominent, with Sweet syndrome being the most frequently reported presentation. Other neutrophilic conditions observed in this context include pustular eruptions and panniculitis (6–11).

Pustular eruptions encompass a broad differential diagnosis, including classic entities such as generalized pustular psoriasis (GPP), drug-induced pustular eruptions, and subcorneal pustular dermatosis (12). Despite the growing recognition of neutrophilic dermatoses as reactive cutaneous manifestations in AOID, pustular eruptions in this context remain inadequately characterized. Key aspects—including detailed rash morphology, systemic associations, laboratory parameters, and patterns of concomitant infection— are not yet fully elucidated. A clearer understanding of these features is essential to improve diagnostic accuracy and inform optimal management strategies. This study aimed to comprehensively characterize the clinical profiles of AOID patients presenting with pustular eruptions and to compare these findings with those observed in patients with GPP. Furthermore, we propose a novel predictive scoring system to aid in the diagnosis of pustular reaction associated with AOID, offering a clinically applicable tool with high discriminative performance.

## 2 Material and methods

### 2.1 Subjects and study design

We performed a retrospective review of clinical data in adult patients (aged >18 years old) with pustular reaction in AOID and GPP in the dermatology clinics of Maharaj Nakorn Chiang Mai Hospital, Chiang Mai, and Srinagarind Hospital, Khon Kaen, Thailand, from January 2014 to June 2023. The diagnosis of AIGA associated with AOID was made according to the following criteria: (i) the positive AIGA using dot enzyme-linked immunosorbent assay (ELISA) (Maharaj Nakorn Chiang Mai Hospital) (13) or via inhibitory ELISA (Srinagarind Hospital) (14); (ii) exclusion of other immunocompromised conditions, including human immunodeficiency virus (HIV), malignancies, or immunosuppressive therapy; and (iii) presence of typical concomitant opportunistic infection resulting from impaired IFN-γ function. Diagnosis of GPP was made by board-certified dermatologists followed the definition proposed in the European Consensus Statement (15). We excluded cases with pustular drug eruption and with non-sterile pustules (evidence of microorganism in pustules from microbiological test). Cases with pustular reaction concomitant with Sweet syndrome were excluded.

We conducted a review of medical records for patients with pustular reactions who attended the dermatology clinic. Clinical manifestations were carefully assessed by the investigators to confirm the diagnosis of pustular reaction in the context of AOID. Demographic data, rash morphology, associated systemic findings, and relevant laboratory results were documented.

Concomitant opportunistic infections in patients with AOID were recorded, including the type of microorganisms, organ involvement, and their temporal relationship to the onset of the pustular reaction. The onset of concomitant infections was defined as the date on which the first clinical symptom or sign of infection—such as fever or lymphadenopathy—was noted.

For patients who experienced multiple episodes of pustular reactions, all episodes were documented. Data collection included both static demographic information for each patient and dynamic records detailing each episode of pustular reaction.

### 2.2 Ethical approval

This study was approved by the Ethics Committee of the Faculty of Medicine, Chiang Mai University (MED-2566-09405) and Khon Kaen University Ethics Committee for Human Research (HE661088).

### 2.3 Statistical analysis

A statistical analysis was conducted using StataNow 18.5 (StataCorp, College Station, Texas, USA) and R version 4.1.2. Data cleaning procedures, including the handling of missing values, removal of duplicates, and standardization of units, were performed prior to analysis. Descriptive statistics included the mean with standard deviation (SD) for continuous data and percentages for categorical data. Independent t-tests were used to compare continuous variables, while Fisher’s exact test was employed for categorical data. Statistical significance was defined as a p-value of less than 0.05.

Variables with more than 50% missing data were excluded from model development. For the development of a predictive equation to estimate the probability of pustular reaction in AOID, variables with significant associations (p< 0.05) in the univariable analysis were included in the multivariable logistic regression. Due to the binary outcome and sparse data, Firth’s logistic regression was used. Missing data were handled with multiple imputations using chained equations (MICE). Variables identified as perfect predictors of the outcome were excluded from model development to avoid exacerbating the sparse data problem. Additionally, patients with these pathognomonic features were not included, as their diagnosis could be easily made, and they were not the target population for our model. We applied backward stepwise elimination, beginning with the full model and sequentially removing variables with p-values greater than 0.05, to identify the most parsimonious set of predictors that remained statistically significant. A predictive model to estimate the probability of pustular reaction was then derived. The area under the receiver operating characteristic (AuROC) curve was calculated to assess discriminative ability. Calibration was evaluated using a calibration plot, expected-to-observed ratio (E:O), calibration slope, and calibration-in-the-large (CITL). The optimal probability cut-off points were determined using Youden’s method.

## 3 Results

### 3.1 Clinical profile of patients with pustular reaction in AOID and GPP

**Figure 1:**
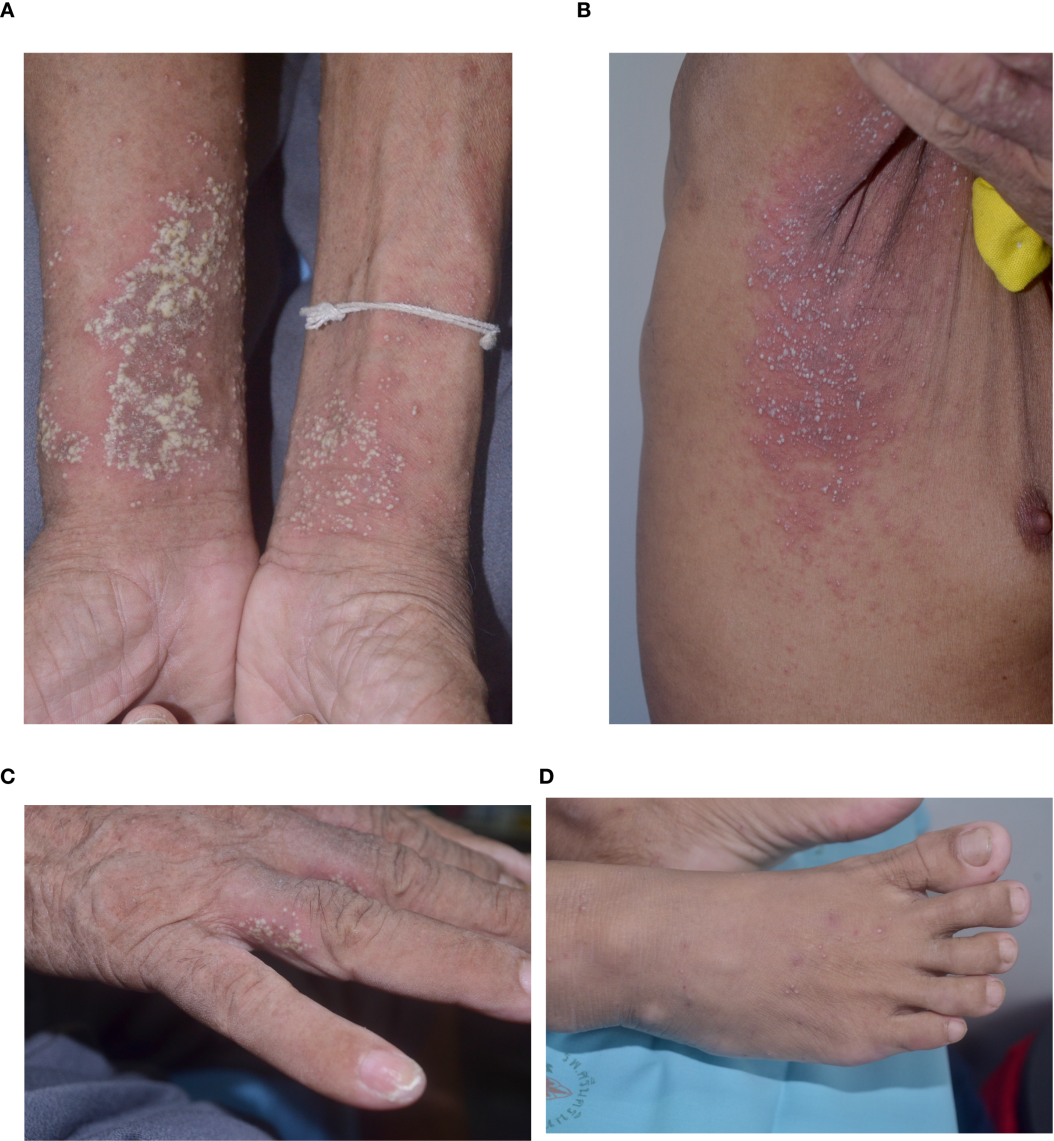
Morphology of lesions in pustular reaction in adult-onset immunodeficiency due to anti-interferon-gamma autoantibody **(A)** volar forearms; **(B)** axilla; **(C)** hand; **(D)** foot.

**Figure 2:**
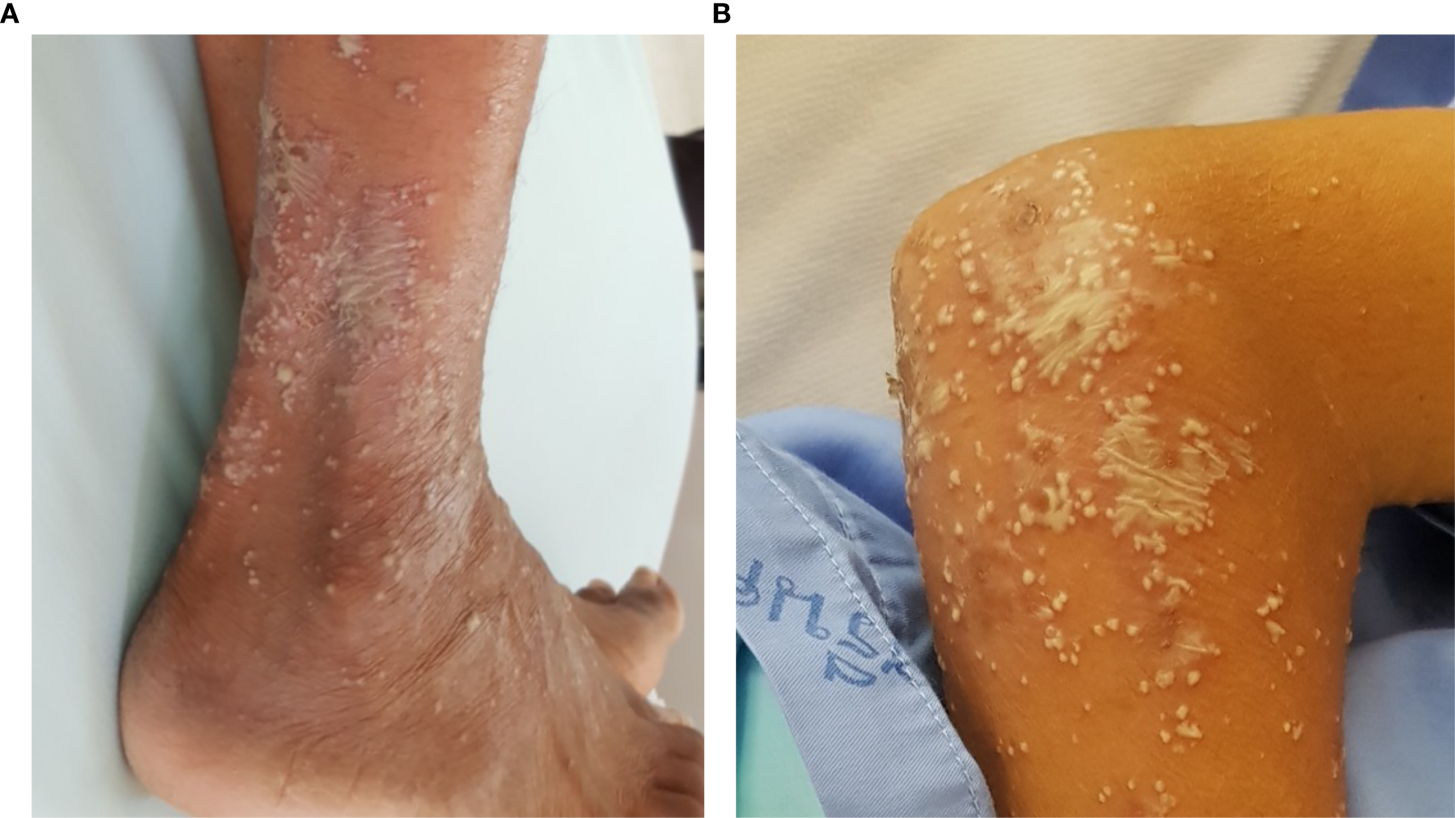
Lake of pus mimicking generalized pustular psoriasis in 2 patients with pustular reaction in adult-onset immunodeficiency due to anti-interferon-gamma autoantibody **(A)** leg; **(B)** forearm.

**Figure 3:**
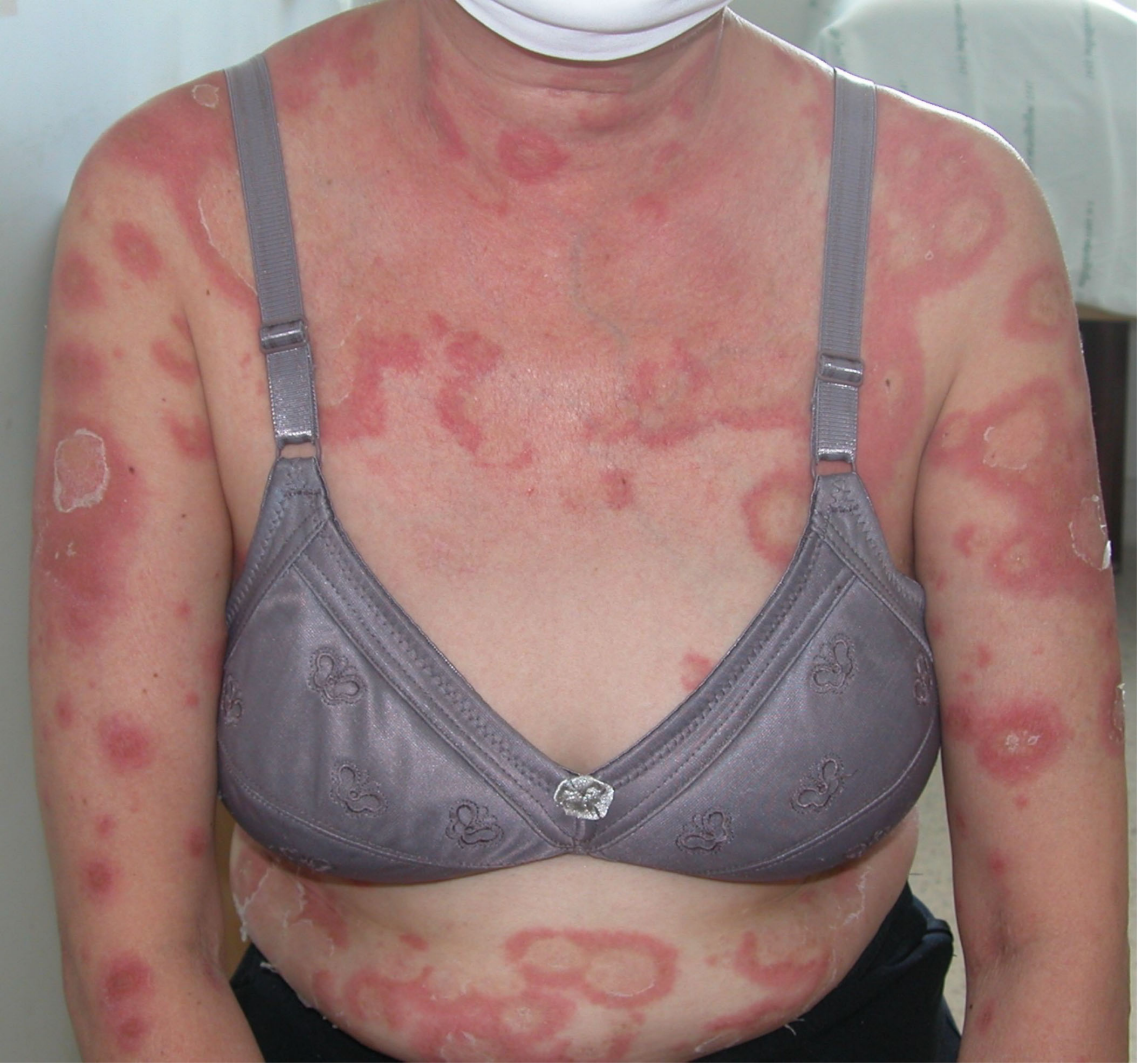
Annular configuration was observed in 1 patient with pustular reaction in adult-onset immunodeficiency due to anti-interferon-gamma autoantibody.

A total of 64 AOID patients with pustular reaction were included in the study (**Table 1**, **Supplementary Figure S1**). There was a slight predominance of females among the patients, with a mean age of 53.2 years. Recurrence of pustular reaction was observed in 18 patients (28.1%). Discrete, pinhead-sized pustules on an erythematous base are characteristic, occasionally coalescing (**Figure 1**). Generalized involvement was found in approximately 60% of the patients. Large confluent pustules, resembling a “lake of pus”, were noted in 2 patients (**Figure 2**), while an annular configuration was observed in 1 patient (**Figure 3**). When compared to a control group of 77 patients with GPP, confluent pustules, annular configuration, geographic tongue, and nail involvement were less frequently observed in pustular reaction of AOID.

**Table 1:** Demographics and clinical characteristics of patients with pustular reaction in AOID and GPP.

Characteristics	Pustular reaction in AOID (n = 64) n (%)	GPP (n = 77) n (%)	*p*-value
Demographic data			
Age (years) (mean ± SD)	53.2 ± 10.3	43.2 ± 17.3	<0.001
Female	38 (59.4)	56 (72.7)	0.108
Alcohol use	12 (25.53%)	5 (9.09%)	0.034
Smoking	5 (10.64%)	2 (3.7%)	0.246
Cutaneous findings			
Symptoms of rash			
Asymptomatic	37 (57.8)	35 (45.5)	0.055
Itch	21 (32.8)	23 (29.9)	
Pain	6 (9.4)	19 (24.7)	
Lake of pus	2 (3.1)	15 (19.5)	0.003
Annular configuration	1 (1.6)	14 (18.2)	0.002
Distribution			
Trunk	31 (48)	40 (52)	0.7
Upper extremities	28 (44)	34 (44)	>0.9
Lower extremities	24 (38)	36 (47)	0.3
Acral areas	21 (33)	17 (22)	0.2
Generalized	38 (59)	59 (77)	0.028
Geographic tongue	0 (0)	6 (7.8)	0.032
Scalp	12 (18.8)	23 (29.9)	0.171
Nail*	0 (0)	20 (26)	<0.001
Recurrent episode of rash	18 (28.1)	26 (33.8)	0.584
Extracutaneous findings			
Concomitant infection	54 (84.4)	6 (7.8)	<0.001
Fever	21 (39.6)	12 (16.9)	0.007
Weight loss	35 (66)	2 (8.7)	<0.001
Arthralgia/arthritis	11 (17.5)	8 (10.4)	0.321
Lymphadenopathy	54 (84.4)	0 (0)	<0.001
Hepatomegaly	10 (15.6)	0 (0)	<0.001
Splenomegaly	6 (9.4)	0 (0)	0.008

AOID, adult-onset immunodeficiency; GPP, generalized pustular psoriasis; *Including pitting nail, onycholysis, subungual pustules, and onychodystrophy.

The most common extracutaneous findings were lymphadenopathy, followed by weight loss, fever, arthralgia/arthritis and hepatomegaly. Compared to GPP, all of these findings, with the exception of arthralgia/arthritis, were significantly more prevalent in the pustular reaction associated with AOID.

Interestingly, we identified several pathognomonic clinical features that serve as strong predictors for each disease. Lymphadenopathy, hepatomegaly, and splenomegaly are indicative of the pustular reaction associated with AOID, while geographic tongue and nail involvement are characteristic of GPP. A total of 61 patients in the cohort did not exhibit any of these pathognomonic features.

### 3.2 Laboratory profile of patients with pustular reaction in AOID and GPP

Anemia, leukocytosis, eosinophilia, thrombocytosis, hyperglobulinemia, and elevated alkaline phosphatase were significantly more common in the pustular reaction associated with AOID compared to GPP (**Table 2**).

**Table 2:** Laboratory characteristics of patients with pustular reaction in AOID and GPP.

Characteristics	Pustular reaction in AOID (n = 64) Mean ± SD	GPP (n = 77) Mean ± SD	*p*-value
Hemoglobin (g/dL)	10.1 ± 1.7	11.8 ± 2	<0.001
MCV (fL)	78.1 ± 8.3	84.5 ± 10.2	0.003
MCH (pg)	24.9 ± 3	28.4 ± 3.8	<0.001
MCHC (g/dL)	31.9 ± 1.1	32.7 ± 1.2	0.012
RDW (%)	16.8 ± 2.7	14.7 ± 1.9	0.001
White blood cell count (x10^3^ cells/μL)	24.1 ± 9.7	13.8 ± 6	<0.001
Neutrophil percentage	75 ± 10.9	75.5 ± 14.2	0.835
Lymphocyte percentage	15.5 ± 7.6	17.1 ± 11.7	0.446
Eosinophil percentage	4.4 ± 4.2	2.1 ± 2.8	0.002
Absolute Neutrophils (x10^3^ cells/μL)	18,980.8 ± 9,219.5	10,973.4 ± 6,008.7	<0.001
Absolute lymphocytes (x10^3^ cells/μL)	3,429.9 ± 1,476	1,922.5 ± 903.7	<0.001
Neutrophil to lymphocyte ratio	8.2 ± 13.6	7.9 ± 7.7	0.877
Absolute eosinophils (x10^3^ cells/μL)	955.9 ± 892.3	246.7 ± 326.7	<0.001
Platelet count (x10^3/^μL)	442.2 ± 155.2	342.3 ± 141.3	0.001
MPV (fL)	8.5 ± 0.9	9.2 ± 1.2	0.050
ESR (mm/hr)	81.3 ± 28.1	87.2 ± 23.9	0.622
Creatinine (mg/dL)	0.8 ± 0.3	0.9 ± 0.4	0.898
Albumin (g/dL)	3.5 ± 2	3.4 ± 0.7	0.748
Globulin (g/dL)	4.9 ± 1.2	3.3 ± 0.8	<0.001
ALT (U/L)	23 ± 12	30.1 ± 22.8	0.058
AST (U/L)	23.3 ± 16.1	40.6 ± 65.1	0.076
ALP (U/L)	198.8 ± 86.2	107.5 ± 50.6	<0.001

AOID, adult-onset immunodeficiency; ALT, alanine transaminase; AST, aspartate aminotransferase; ALP, alkaline phosphatase; ESR, erythrocyte sedimentation rate; GPP, generalized pustular psoriasis; MCH, mean corpuscular hemoglobin; MCHC, mean corpuscular hemoglobin concentration; MCV, mean corpuscular volume; MPV, mean platelet volume; RDW, red cell distribution width.

### 3.3 Concomitant infections of patients with pustular reaction in AOID and GPP

Concomitant infections were identified in 54 cases (84.4%) among AOID patients with pustular reaction (**Table 3**). A total of 79 episodes of pustular reaction associated with concomitant infections were documented. In the majority of episodes (n = 64), the infection preceded the onset of the pustular reaction, with a median interval of 31 days. Conversely, in 10 episodes, the infection occurred subsequent to the pustular reaction, with a median interval of 18 days following the onset of the dermatologic manifestation.

**Table 3:** Concomitant infections related to onset of pustular reaction in AOID patients (all episodes).

Characteristics	Total episodes (n = 79) n (%)	Infection preceding reactive pustular reaction (n = 64) n (%)	Infection following reactive pustular reaction (n =10) n (%)	Unidentified Infection onset (n = 5) n (%)
Interval to onset of pustular reaction in days, median (IQR)	24 (0, 103)	31 (4.75, 140)	18 (3.5, 28.5)	0 (0, 0)
Organisms				
Mycobacteria	70 (88.6)	55 (85.9)	10 (100)	5 (100)
NTM				
RGM	30 (38)	24 (37.3)	5 (50)	1 (20)
SGM	4 (5.1)	4 (6.2)	0 (0)	0 (0)
Unclassified NTM	12 (15.2)	8 (12)	3 (30)	1 (20)
Suspected NTM	20 (25.3)	16 (25)	2 (20)	2 (40)
Mycobacterium tuberculosis	4 (5.1)	3 (4.7)	0 (0)	1 (20)
Bacteria	8 (10.1)	8 (12)	0 (0)	0 (0)
Salmonella	6 (7.6)	6 (9.4)	0 (0)	0 (0)
* Burkholderia cepacia*	2 (2.5)	2 (3.1)	0 (0)	0 (0)
Fungi	1 (1.3)	1 (1.6)	0 (0)	0 (0)
*Cryptococcus*	1 (1.3)	1 (1.6)	0 (0)	0 (0)
Organ involvement				
Lymph node	67 (84.8)	52 (81)	10 (100)	5 (100)
Blood	6 (7.6)	6 (9.4)	0 (0)	0 (0)
Joint	3 (3.8)	2 (3.1)	1 (10)	0 (0)
Bone	2 (2.5)	2 (3.1)	0 (0)	0 (0)
Central nervous system	1 (1.3)	1 (1.6)	0 (0)	0 (0)
Lung	5 (6.3)	5 (7.8)	0 (0)	0 (0)
Liver/spleen	3 (3.8)	2 (3.1)	1 (10)	0 (0)
Skin	3 (3.8)	3 (4.7)	0 (0)	0 (0)

AOID, adult-onset immunodeficiency; NTM, non-tuberculous mycobacterium; RGM, rapid growing mycobacterium; SGM, slow growing mycobacterium.

Among all documented episodes, the most frequently identified concomitant infections were caused by rapidly growing mycobacteria. The three most commonly involved organ systems were the lymph nodes, bloodstream, and lungs.

In patients with GPP, concomitant infections were observed in 6 of 77 individuals. When analyzed by episode, infections were implicated as potential triggers in 11 of 149 episodes of GPP flares. Identified infectious triggers included respiratory viral infections (such as common cold and SARS-CoV-2), bacterial infections (including erysipelas and *Staphylococcus aureus* pyoderma in 2 cases), and chronic hepatitis C virus infection.

### 3.4 Predictive scoring system

After removal of pathognomonic variables, the rest variables with significant association (p< 0.05) from univariable logistic regression were added into multivariable logistic regression as a full model (**Table 4**). After stepwise backward elimination, the remaining significant variables in final reduced model were concomitant infection (OR 57.98; 95% CI 12.45, 270.04), globulin level in g/dL (OR 6.39; 95% CI 2.16, 18.91) and each 10 U/L increment of alkaline phosphatase level (OR 1.24; 95% CI 1.07, 1.45). The formula to predict probability of pustular reaction is as follows:

**Table 4:** Multivariable logistic regression (full model and reduced model).

Predictors	Full model		Reduced model		β	Bootstrap-corrected β
	OR (95%CI)	*p*-value	OR (95%CI)	*p*-value		
Age (per 10 year)	2.91 (0.74, 11.50)	0.128	Not included			
Alcohol use	5.73 (0.09, 379.53)	0.281	Not included			
Lake of pus	0.00 (0.00, 3.53)	0.111	Not included			
Annular configuration	1.20 (0.03, 50.45)	0.467	Not included			
Generalized involvement	0.08 (0.00, 1.38)	0.082	Not included			
Concomitant infection	1298.48 (11.72, 143869.5)	0.003	57.98 (12.45, 270.04)	<0.001	4.0601	1.5266
Fever	1.79 (0.09, 37.59)	0.708	Not included			
Weight loss	0.34 (0.01, 14.69)	0.577	Not included			
Haemoglobin (per 1 g/dL)	2.46 (0.80, 7.55)	0.116	Not included			
MCV	0.89 (0.72, 1.09)	0.250	Not included			
WBC count (per 1,000)	1.17 (0.94, 1.46)	0.157	Not included			
Eosinophils percentage	1.03 (0.72, 1.46)	0.888	Not included			
Platelet count	1.00 (0.99, 1.01)	0.939	Not included			
Globulin (g/dL)	4.43 (0.46, 42.55)	0.198	6.39 (2.16, 18.91)	0.001	1.8546	0.6973
ALP (per 10 U/L)	1.35 (0.98, 1.88)	0.069	1.24 (1.07, 1.45)	0.005	0.2173	0.0817
Constant					-12.4355	-4.8900

ALP, alkaline phosphatase; MCV, mean corpuscular volume; WBC, white blood cell.


$$\mathrm{Pr}\left(Reactive\;pustular\;reaction\right)=\frac{e^{-12.4355+\left(\left(\frac{ALP}{10}\right)\times 0.2173\right)+\left(CI\times 4.0601\right)+\left(GLOB\times 1.8546\right)}}{1+e^{-12.4355+\left(\left(\frac{ALP}{10}\right)\times 0.2173\right)+\left(CI\times 4.0601\right)+\left(GLOB\times 1.8546\right)}}$$

Where CI is presence of concomitant infection, GLOB is globulin level (g/dL) and ALP is alkaline phosphatase level (U/L). A calculator for this formula is available at the following link: https://cmu.to/pustular-risk.

**Figure 4:**
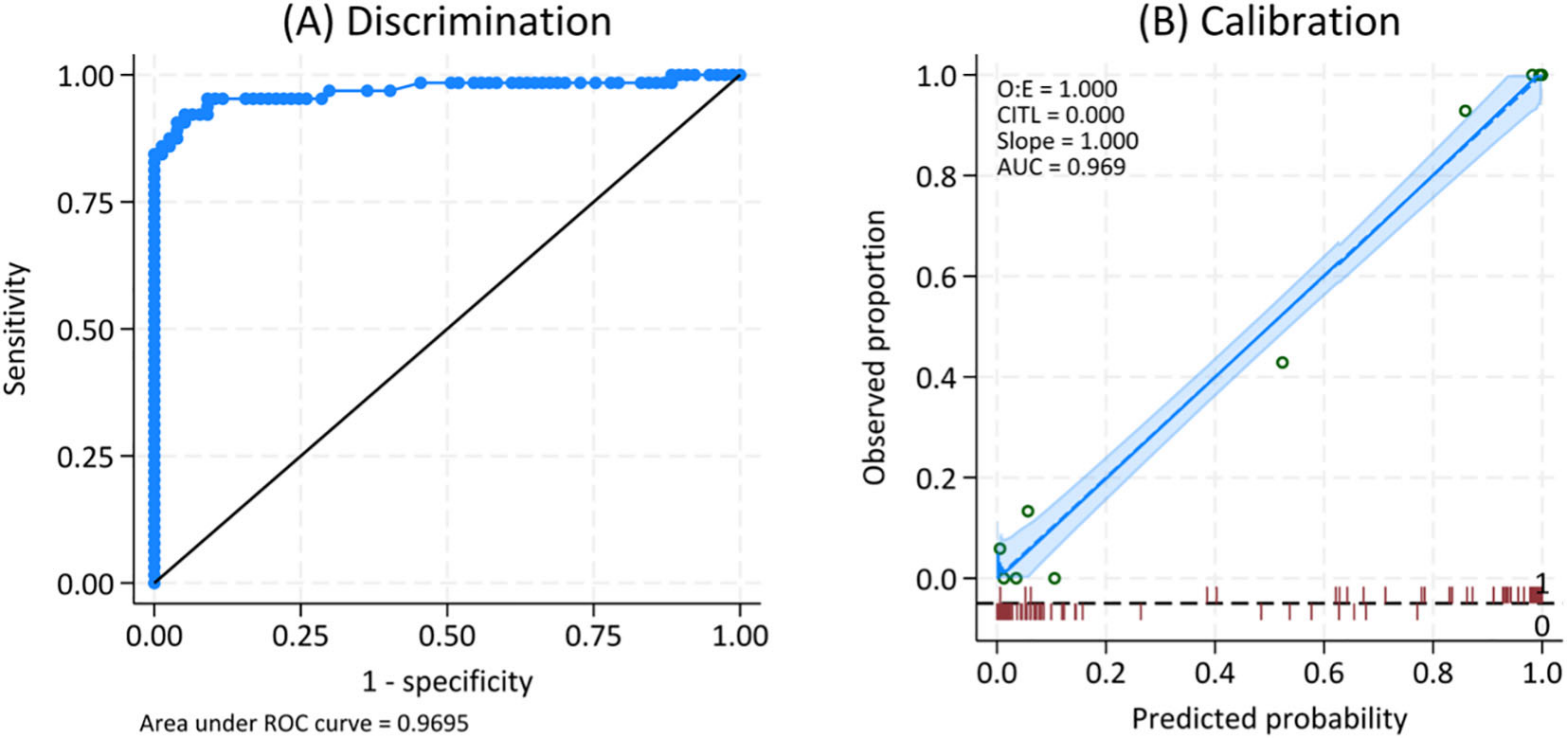
ROC **(A)** and calibration curve **(B)** for the predicted probability of pustular reaction in adult-onset immunodeficiency due to anti-interferon-gamma autoantibody.

When applying this equation to all 141 patients, we found the outstanding discriminative ability with AuROC 0.97; 95% CI 0.94, 1.00 and almost perfect calibration curve (**Figure 4**). The best cut point for the predicted risk was 60%, with a sensitivity of 92% and a specificity of 95%. In comparison, a model including only concomitant infection yielded a lower AuROC of 0.88 (95% CI: 0.83–0.94), indicating that the inclusion of globulin and alkaline phosphatase improved the model’s predictive accuracy (P<0.001).

Additionally, when this equation was applied to 61 patients lacking pathognomonic variables, it demonstrated a discriminative ability with AuROC 0.78; 95% CI: 0.55–1.00. The optimal cut-off point for the predicted risk was 32%, yielding a sensitivity of 62% and a specificity of 91%.

## 4 Discussion

To date, pustular reaction in AOID has not been the focus of a dedicated investigation, in contrast to Sweet syndrome, which is more extensively documented. This study represents the first attempt to delineate the clinical features, systemic associations, and diagnostic considerations of this distinct neutrophilic dermatosis within the AOID spectrum.

The pustular reaction observed in AOID is clinically characterized by an acute eruption of pinhead-sized pustules on an erythematous base. Histopathological examination typically reveals subcorneal and/or intraepidermal pustules (9). To date, the pathogenesis of pustular eruptions in the context of AOID remains poorly elucidated. However, it is plausible that these cutaneous manifestations represent a subset within the broader spectrum of reactive neutrophilic dermatoses. Such eruptions are likely driven by aberrant crosstalk between the innate and adaptive immune systems, with particular involvement of the T helper 1 and T helper 17 pathways. The signature cytokines of these respective axes are IFN-γ and interleukin-17 (16). Despite increasing recognition of this entity, a standardized nomenclature has yet to be established. Various terms have been employed in prior literature to describe this reaction, including exanthematous pustulosis, acute generalized exanthematous pustulosis (AGEP), pustular psoriasis, and generalized pustular reaction (1, 6, 7, 9, 11, 17).

However, the term AGEP may be suboptimal for describing pustular reaction in the context of AOID for several reasons. Firstly, in our cohort, only 51 of 94 episodes (54.3%) exhibited a truly generalized distribution, thereby challenging the appropriateness of the term “generalized” in this clinical setting. Furthermore, over 90% of AGEP cases reported in the literature are attributed to drug-induced etiologies, with viral infections representing the predominant cause among the non-drug-related cases. Importantly, to date, no instances of AGEP secondary to *Mycobacterium* infection have been documented, further underscoring the potential limitations of applying this nomenclature in AOID-associated presentations.

We observed that a considerable number of AOID cases presenting with concomitant infection were subsequently treated with antibiotics or anti-tuberculosis agents, after which pustular eruptions developed. These eruptions were frequently diagnosed as drug-induced AGEP. However, a significant proportion of such cases may represent pustular reaction intrinsic to AOID rather than true drug-induced AGEP. This diagnostic ambiguity likely contributes to the under-recognition and underreporting of pustular reaction associated with AOID.

Differentiating the pustular reaction associated with AOID from GPP remains a clinical challenge. Dermatologic assessment traditionally emphasizes features such as annular configuration and the presence of a “lake of pus” as suggestive of GPP. However, these features are not pathognomonic. In our cohort, one case of AOID-associated pustular reaction exhibited an annular configuration, and two cases demonstrated a lake of pus, indicating that these findings may also occur in the context of AOID. Thus, reliance on these clinical signs alone may be insufficient for accurate diagnostic distinction between the two entities. Recently, mutations in **SERPINA1**, **SERPINB3**, and **TGFBR2** have been identified in both patients GPP and those with AOID-associated pustular reaction, suggesting a potentially shared pathogenic mechanism between the two conditions (18–20) Based on these findings, we propose that GPP-like lesions may represent a clinical variant of the pustular reaction observed in AOID.

In patients presenting with a pustular eruption, following the exclusion of drug-induced eruptions and pyogenic skin infections, several distinguishing clinical clues may aid in differentiating the pustular reaction associated with AOID from GPP. Findings from this study underscore the importance of a thorough physical examination. Specifically, targeted evaluation of the nails, tongue, lymph nodes, liver, and spleen is recommended, as nail abnormalities and geographic tongue are more indicative of GPP, whereas the presence of lymphadenopathy and hepatosplenomegaly favors a diagnosis of AOID-associated pustular reaction. In cases where these clinical features are absent or inconclusive, a predictive scoring system may be utilized to estimate the likelihood of AOID-associated pustular disease, thereby aiding diagnostic decision-making. The presence of concomitant infection, along with elevated serum globulin and alkaline phosphatase levels, further strengthens the clinical suspicion of AOID-associated pustular reaction. Moreover, recently published studies have identified specific human leukocyte antigen (HLA) alleles—most notably *HLA-DPB1*05:01 and *HLA-DRB1*15:02—as potential genetic biomarkers capable of distinguishing pustular eruptions associated with AOID from GPP (21).

Involvement of the nails and tongue in GPP reflects systemic inflammation characteristic of mucocutaneous psoriasis. Geographic tongue, in particular, shares histopathological features with cutaneous psoriasis. Notably, the higher prevalence of pustules of Kogoj observed in geographic tongue further supports its association with pustular psoriasis and underscores its potential as a clinical marker in differentiating GPP from other pustular dermatoses (22).

The involvement of the reticuloendothelial system, including lymphadenopathy and hepatosplenomegaly, correlates with the presence of concomitant infections in AOID-associated pustular reaction, emphasizing its role as a critical indicator. In conjunction with elevated globulin and alkaline phosphatase levels, this systemic involvement supports the use of these markers as key predictors in predictive models for identifying AOID-related pustular reaction. Previous studies have demonstrated significantly higher alkaline phosphatase levels in AOID patients compared to both healthy individuals and HIV-infected patients. Moreover, within the same cohort of AOID patients, alkaline phosphatase levels are notably higher during active infections compared to periods without infection, further underscoring its utility as a dynamic biomarker of disease activity (5).

Our findings regarding concomitant infection in AOID-associated pustular reaction are consistent with previous studies on AOID-associated Sweet syndrome, which also identified rapidly growing mycobacteria as the most prevalent pathogens (23). Based on the shared clinical and immunological context—namely, the occurrence of neutrophilic dermatoses in the setting of AOID—we propose a potential interaction between AOID, NTM infection, and the development of pustular reaction. This suggests a common pathogenic mechanism linking the presence of AIGA, microbial triggers, and neutrophilic skin inflammation.

Regarding the temporal relationship between concomitant infection onset and pustular reaction, most pustular reactions in AOID occurred after the onset of concomitant infection; however, a substantial number of cases presented before active infection. This finding highlights the importance of clinicians actively screening for concomitant infection in AOID patients experiencing skin flare-ups.

Currently, no established guidelines exist for the treatment of reactive dermatoses in this syndrome. The majority of reported treatments primarily rely on corticosteroids (9). Additionally, acitretin has emerged as an alternative therapeutic option for pustular reaction, demonstrating satisfactory outcomes in affected patients (24).

This study has some limitations. First, the study is inherently retrospective in nature, which may introduce recall bias or misinterpretation of data. This could affect the accuracy of patient histories and clinical assessments. Additionally, the prevalence of pustular reaction in AOID may be underestimated, as some cases may have been misdiagnosed as drug-induced AGEP, particularly in those with a history of drug exposure. However, in this study, we excluded cases with a clear history of drug-induced AGEP based on drug exposure and temporal associations. Future prospective studies focusing on distinguishing pustular reaction in AOID from AGEP may provide valuable insights. Thirdly, detailed histopathological data were not available, and such features may offer additional clues to aid in differentiating these two conditions. Finally, the algorithm developed in this study was derived exclusively from a cohort of patients with AOID and GPP. Its performance and specificity in distinguishing AOID-associated pustular reactions from other pustular dermatoses or pustular eruptions occurring in immunodeficiencies of different etiologies (e.g., HIV infection, primary immunodeficiency syndromes, or iatrogenic immunosuppression) have not yet been validated.

## 5 Conclusion

This is the first study to specifically focus on pustular reaction in AOID. Clinically, pustular reaction in AOID may be difficult to distinguish from GPP, as both conditions can present with confluent pustules—often referred to as “lakes of pus”—and annular configurations, which are considered signature features of GPP. It must be emphasized, however, that such hallmark morphologies are only rarely and inconsistently observed in AOID-associated pustular reactions. We identified several pathognomonic clinical clues that may help differentiate AOID-associated pustular reaction from GPP, including lymphadenopathy, hepatomegaly, and splenomegaly. Nevertheless, in the absence of these features, a combination of concomitant infections, elevated globulin levels, and alkaline phosphatase levels can be utilized to estimate the likelihood of pustular reaction in AOID.

## Data Availability

The original contributions presented in the study are included in the article/**Supplementary Material**. Further inquiries can be directed to the corresponding authors.

## Supplementary material

Supplementary Figure 1 Number of participants from each institution.
