# Supplementary figures and images for: Pustular reaction in adult-onset immunodeficiency due to anti-interferon-gamma autoantibodies
Source: Front Immunol. 2025 Aug 22;16:1619832. doi: 10.3389/fimmu.2025.1619832 (PMC12411423; doi:10.3389/fimmu.2025.1619832)

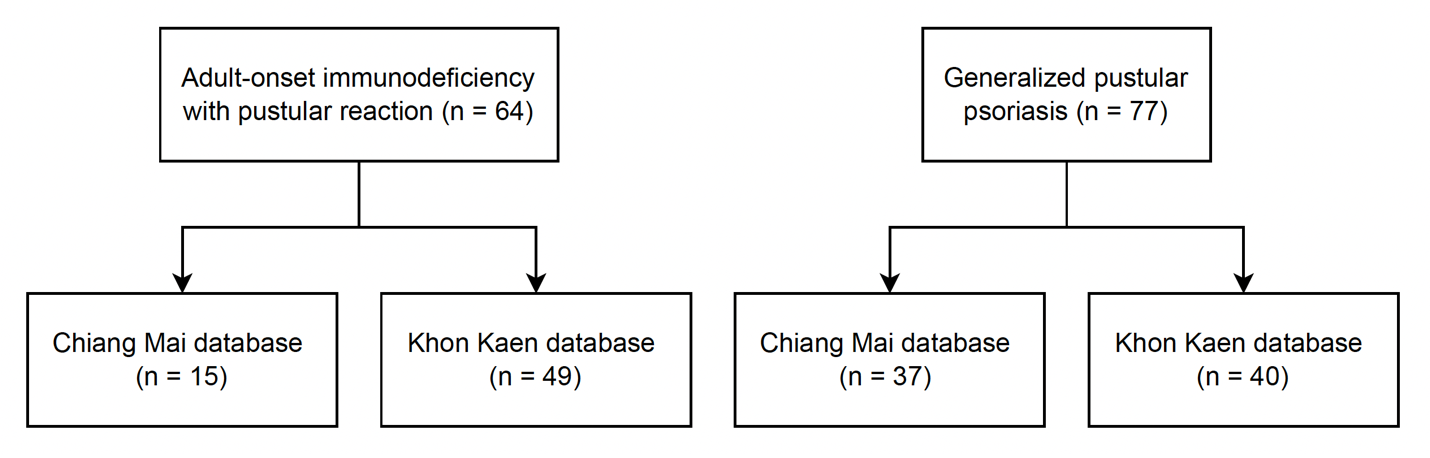

Supplement: Supplementary Figure 1 — Number of participants from each institution. [file Image1.tiff]
